# The Effect of Dental Treatments in Caries Management on Stress and Salivary Protein Levels

**DOI:** 10.3390/jcm11154350

**Published:** 2022-07-27

**Authors:** Raluca-Paula Vacaru, Andreea Cristiana Didilescu, Ruxandra Sfeatcu, Mihaela Tănase, Aneta Munteanu, Daniela Miricescu, Wendy Esmeralda Kaman, Hendrik Simon Brand

**Affiliations:** 1 Division of Embryology, Faculty of Dental Medicine, Carol Davila University of Medicine and Pharmacy, 8 Eroii Sanitari Boulevard, 050474 Bucharest, Romania; raluca.vacaru@umfcd.ro; 2 Division of Pedodontics, Faculty of Dental Medicine, Carol Davila University of Medicine and Pharmacy, 8 Barajul Iezeru, 032799 Bucharest, Romania; mihaela.tanase@umfcd.ro (M.T.); aneta.munteanu@umfcd.ro (A.M.); 3 Department of Oral Biochemistry, Academic Centre for Dentistry Amsterdam (ACTA), VU University of Amsterdam and University of Amsterdam, Gustav Mahlerlaan 3004, 1081 LA Amsterdam, The Netherlands; w.e.kaman@acta.nl (W.E.K.); h.brand@acta.nl (H.S.B.); 4 Division of Oral Health and Community Dentistry, Faculty of Dental Medicine, Carol Davila University Medicine and Pharmacy, 17–21 Calea Plevnei Street, Sector 1, 010221 Bucharest, Romania; 5 Division of Biochemistry, Faculty of Dental Medicine, Carol Davila University of Medicine and Pharmacy, 8 Eroii Sanitari Boulevard, 050474 Bucharest, Romania; daniela.miricescu@umfcd.ro

**Keywords:** anxiety, dental caries, detection and diagnosis, salivary alpha-amylase, salivary cortisol, psychometric evaluations, treatment

## Abstract

A great burden is put on healthcare systems by dental caries and understanding patients’ treatment needs is of utmost importance. The aim of this pre–post study was to assess dental anxiety and the psychological stress induced by two different types of dental treatment (prophylaxis and cavity preparation), by combining psychometric evaluations with salivary biomarkers, in a group of 28 schoolchildren presenting in a university clinic. Pre- and post-treatment unstimulated whole saliva was collected and levels of cortisol, alpha-amylase (sAA) and total protein content were measured. The State–Trait Anxiety Inventory for Children and the Frankl Behaviour Rating Scale (FBRS) were applied. Statistical analysis was performed using the Stata/IC 16 (StataCorp) programme. All salivary parameters showed strong positive correlations between pre- and post-treatment levels. Post-treatment, salivary cortisol decreased (*p* = 0.008, paired *t*-test), sAA did not change significantly (*p* = 0.572, sign test), while the sAA/cortisol ratio (AOC) increased (*p* = 0.036, sign test). There were no correlations between state and trait anxiety levels. State anxiety scores registered significantly higher values for children with an FBRS score of 3 compared with a score of 4 (*p* < 0.001, unpaired *t*-test). The post-treatment decrease in the salivary cortisol level was higher for prophylaxis compared with the cavity preparation group (*p* = 0.024, *t*-test). These results demonstrate that sAA and cortisol levels are altered differently by psychological stress induced by two different types of dental treatment.

## 1. Introduction

Dental caries is a progressive and irreversible pathology occurring as a consequence of an imbalance that arises between protective and cariogenic factors in the oral cavity [1]. This chronic disease interests the whole population, irrespective of age, affecting both primary and permanent dentitions. Disease severity increases progressively at older ages. This widespread disease poses a great burden on healthcare systems, as costs of treatments represent 5% to 15% of their budgets [2,3]. From 2005 to 2015, dental caries incidences had increased globally, with 14.2% in permanent teeth and with 5.6% in deciduous teeth [2].

On the one hand, dental caries had been shown to be an influential factor in the occurrence of dental anxiety, especially if the patients were subjected to painful symptomatology or other unfavourable consequences. Unfavourable consequences such as negatively affected general health and development, poor masticatory efficiency, altered nutrition and unaesthetic appearance might consequently lead to social inequalities as well as poor school or work performances, which could further enhance anxiety levels [4,5]. On the other hand, stress and other psychosocial factors had been incriminated in aggravating dental disease [6,7]. In fact, children experiencing dental anxiety tend to access dental services less frequently and are less predictable regarding their visits, which further contributes to their oral health problems [8]. Therefore, it is necessary to promptly identify any signs of dental anxiety in children and approach them with suitable behavioural management techniques to reduce the level of dental anxiety or at least prevent worsening of the situation. A dentist’s clinical experience and perception of a child’s behaviour are not sufficient for correctly assessing an anxious patient; it has been shown that there are discrepancies between dentists’ and children’s scoring on tests evaluating dental anxiety [9]. Furthermore, many dentists do not realize how dental treatments may impact their patients’ psychological wellbeing and how stressful dental visits can actually be [10]. Moreover, parents may also experience dental anxiety, which they transmit upon their children, frequently involuntarily [11]. 

Dental anxiety most frequently occurs in childhood and adolescence and can be considered as a form of stress [12,13]. A recently published systematic review showed that 36.5% of preschool children, 25.8% of schoolchildren and 13.3% of adolescents were experiencing dental anxiety [14]. When exposed to a stressful situation, two reaction systems are activated to help the organism to adapt and cope: the hypothalamic–pituitary–adrenal system (HPA axis), which releases cortisol, and the autonomic nervous system (ANS), which comprises both the sympathetic–adrenal–medullary system and the parasympathetic system, subsequently releasing catecholamines [15]. Excessive stimulation of these systems may lead to deregulations, which affect emotional, cognitive, metabolic and immune systems [16]. 

Behavioural medicine considers cortisol to be the “golden standard” biomarker for stress [17]. Cortisol is excreted in saliva and its levels correspond to serum concentrations, independently of salivary flow rate [18], reliably reflecting its changes throughout the day [19]. Measurement of salivary cortisol has the additional advantage of non-invasive collection, as opposed to venepuncture, a method which might be considered a stressor itself [20]. 

Similarly, salivary alpha-amylase (sAA) has been recognised and validated as a reliable and specific marker for ANS activity and has been proposed as an alternative biomarker for stress [21,22]. sAA is biologically correlated with adrenaline and noradrenaline, but independent of their plasma levels [23,24], although available evidence is still inconclusive [25,26]. In fact, the oral cavity’s equilibrium is maintained by saliva [27], and as salivary glands’ functionality is under ANS’s regulatory action, psychological stress might influence both saliva’s secretion and composition, and indirectly also bacterial aggregation, thereby affecting its protective capacity against the development and progression of dental caries [28,29,30]. Moreover, chronic stress has different effects on the ANS and HPA axis, sAA showing a higher sensitivity than cortisol, and being able to differentiate chronic stress-affected patients from their controls [31].

In addition to the measurement of hormones levels, there are a variety of instruments that have been proposed for the evaluation of anxiety in the dental office that are able to assess children’s responses to dental procedures. These include self-reported psychometric questionnaires such as the State–Trait Anxiety Inventory for Children [32], and observational behavioural scales such as the Frankl Behaviour Rating Scale [33]. These scales can also improve the communication between child, parent and dentist by acknowledging the child’s specific concerns and necessities [34].

Therefore, understanding, detecting and efficiently managing dental anxiety in children is imperative for a paediatric dentist. This should be achieved in a practical manner that allows clinical practitioners to include this in their daily practice in order to effectively improve their behavioural management approach. Our study aims to assess dental anxiety in a group of schoolchildren presenting for dental treatment in a specialised paediatric dentistry clinic and to evaluate the stress induced by two different dental treatments, by combining psychometric evaluations with measurements of salivary stress protein levels.

## 2. Materials and Methods

### 2.1. Participants

Twenty-eight physically and mentally healthy children, aged 5 to 13 years old, who presented for the first time at the Paediatric Dentistry Clinic of the Faculty of Dental Medicine, Carol Davila University of Medicine and Pharmacy, Bucharest between 2019 and 2022, were initially recruited for the study. This study was approved by the Research Ethics Committee at Carol Davila University of Medicine and Pharmacy (no. 188/28 January 2019). Before enrolment, a written informed consent was signed by the parents or legal guardians of the participants after they received information about the aims and protocols of the study. The participants were eligible for inclusion in the study if they did not have a history of systemic diseases or mental disabilities and did not receive any medication in the last 3 months that might affect salivary gland functioning or cortisol and sAA levels. The presence of intraoral lesions was another reason for exclusion. 

The participants were recruited from patients presenting in the clinic for the first time. We included all the patients who met the eligibility criteria and whose parents or legal guardian agreed to their participation. A priori sample size calculation was performed using G*Power software, version 3.1.9.4 (Heinrich-Heine-Universität Düsseldorf, Düsseldorf, Germany) based on the results of our previous studies on cortisol levels in whole saliva [35]. A change in salivary cortisol levels of 2 ng/mL would correspond with an effect size of 0.5. Based on an effect size of 0.5, an α of 0.05 and a power of 80%, a minimum of 27 participants was needed for a pre/post comparison of the cortisol concentration in the total study population.

### 2.2. Study Design

In this pre–post study, standard clinical examinations and dental interventions were performed by a single trained examiner (R.-P.V.) at a dental office. Before enrolment in the study, the children and their parents or legal guardians were informed about the study, documents were filled and signed, and afterwards initial evaluations were performed. Written instructions regarding the necessary conditions for saliva collection were provided and explained. Two samples of unstimulated whole saliva were collected per subject, one before and another one 30 min after finalisation of the intervention. Fourteen children received a prophylactic treatment consisting of professional brushing with rotary instruments and topical fluoridation using fluoride gels. Fourteen children received restorative dental treatment, consisting of a cavity preparation that could be conducted without the need to perform pain control through local anaesthesia; thus, the stress experience was minimal and could not be generated by needle phobia. After the dental treatment was finished, the children were asked to fill a psychometric test—State–Trait Anxiety Inventory for Children—under the supervision and guidance of the paediatric dentist (R.-P.V.). Nine of the subjects had previously received dental treatment at another dental office by a dentist not included in the present study. The parents and patients did not relate any traumatic experiences.

### 2.3. Saliva Collection

For standardisation reasons, all interventions and saliva collections were scheduled between 8 AM and 11 AM in order to reduce diurnal changes of cortisol and sAA levels. Both cortisol and sAA have distinct diurnal profiles; cortisol reaches peak values within half an hour after waking up and afterward drops throughout the day, as opposed to sAA, which drops heavily upon waking up and then rises throughout the day [36,37]. The conditions for saliva collection were: no consumption of food, liquids or chewing gum for at least 2 h prior to saliva collection and no oral hygiene measures performed in the morning. Furthermore, in order to reduce potential confounding effects of physical stress, the subjects were recommended to not perform any physical effort, such as running, prior to arriving at the dental clinic. In the treatment room, the children were seated in the dental chair and asked to transfer saliva pooling on their mouth floor into the sterile tubes provided by using the passive drooling method [38]. During saliva collection, the subjects remained calm and abstained from exhibiting any excessive orofacial movements, as well as swallowing the saliva or speaking. Saliva samples were centrifuged at 3500× *g* for 15 min and saliva supernatants were stored at −80 °C until biochemical analysis.

### 2.4. Biochemical Analyses

Salivary cortisol was assessed using an ELISA method with a commercially available kit (NovaTec Immundiagnostica GmbH, REF DSNOV20, Dietzenbach, Germany), following the manufacturer’s instructions. In brief, 25 μL of saliva were incubated with 200 μL of Cortisol-HRP Conjugate for 1 h at 37 °C. Afterwards, 100 μL TMB substrate solution was added and after 15 min the process was stopped by adding 100 μL of stop solution. Salivary cortisol was detected using a Dialab microplate reader and the results were expressed in ng/mL. Salivary α–amylase activity was determined with a colorimetric-based enzymatic activity assay using an amylase specific substrate (2-chloro-4-nitrophenyl-α-D-maltotrioside, Sigma-Aldrich, Zwijndrecht, The Netherlands) as previously described [39] and the results were expressed in U/mL. Total protein content was measured using the Pierce™ BCA Protein Assay Kit (Thermo Scientific, Landsmeer, The Netherlands) as described previously [39] and the results were expressed in µg/mL. All biochemical measurements were performed in duplicate.

### 2.5. Psychometrical Analyses

The validated Romanian version of the State–Trait Anxiety Inventory for Children scale (Mindgarden, Inc., Menlo Park, CA, USA, licence OL-00008675/ 2019-11.22 distributed by D&D Consultants/TestCentral) was applied for anxiety assessments. The test consisted of two forms, each comprising 20 items, which evaluated how the subject was feeling. The “state” anxiety form (STAIC S-Anxiety) is an indicator of how the subject is feeling as a consequence of a specific situation which might provoke acute stress, such as the dental treatment related to our study. The “trait” anxiety form (STAIC T-Anxiety) determines the impact of general situations and contexts over anxiety levels, picturing a more comprehensive measure of anxiety. The questionnaires were filled under the supervision and guidance of the paediatric dentist in the same operatory room where the treatments were performed, immediately after the treatment and before the collection of the post-treatment saliva sample. The scoring was performed both manually, following the indications in the guidance manual, and electronically, using the on-line platform, provided by D&D Consultants/TestCentral. Further processing of the raw scores was electronically performed on the platform; the scores were normalised to the Romanian population, corresponding to the subject’s sex and grade (normalised T-scores), and the results were divided automatically into low, moderate and high anxiety levels.

### 2.6. Observational and Behavioural Rating

Situational fear was subjectively measured by using the Frankl Behaviour Rating Scale (FBRS). The child’s reaction to dental treatment was assessed by the paediatric dentist performing the intervention (R.-P.V.) in one of the following categories of behaviour: definitely negative (1), negative (2), positive (3) and definitely positive (4) [33].

### 2.7. Data Analysis

Statistical data processing was performed using the program Stata/IC 16 (StataCorp. 2019. Stata Statistical Software: Release 16. College Station, TX, USA: StataCorp LLC). A Shapiro–Wilk test was performed to test normal distribution. Data distributions were expressed as means, standard deviations (SD), medians and percentages. Comparisons between pre-treatment and post-treatment levels for the evaluated salivary parameters were determined using a paired *t*-test or a sign test. Intergroup comparisons were performed using an unpaired *t*-test. Correlations between variables were investigated using either Pearson (*r*) or Spearman (*r_s_*) correlation coefficients. Statistical significance was considered for *p*-values < 0.05.

## 3. Results

The study group consisted of 28 children with a mean (SD) age of 8.03 (2.12) years old, of whom 7 were boys (25%) and 21 were girls (75%). All subjects presented untreated dental caries in both permanent and primary teeth, with a mean (SD) number of 7.86 (4.65) teeth decayed per subject. The mean (SD) number of teeth present in the oral cavity per subject were 23.36 (2.57), with a minimum of 17 teeth and a maximum of 28 teeth present, of which 33.87 ± 20.34% of the teeth per subject were affected by untreated dental caries. The mean (SD) scores for carious experience indices were: DMFT = 2.77 (3.08), DMFS = 3.96 (4.31), dmft = 6.88 (4.44), dmfs = 17.72 (14.86).

Salivary evaluations presented a wide range variability and therefore we normalised salivary cortisol and alpha-amylase levels to total protein content. Furthermore, the ratio of sAA over cortisol (AOC) was calculated as a combined marker of both stress systems’ responses [40]. The mean and standard deviations of the raw and normalised levels of the evaluated salivary parameters are presented in Table 1. Comparison between pre-treatment and post-treatment levels showed statistically significant differences only for salivary cortisol and AOC assessments.

**Figure 1:**
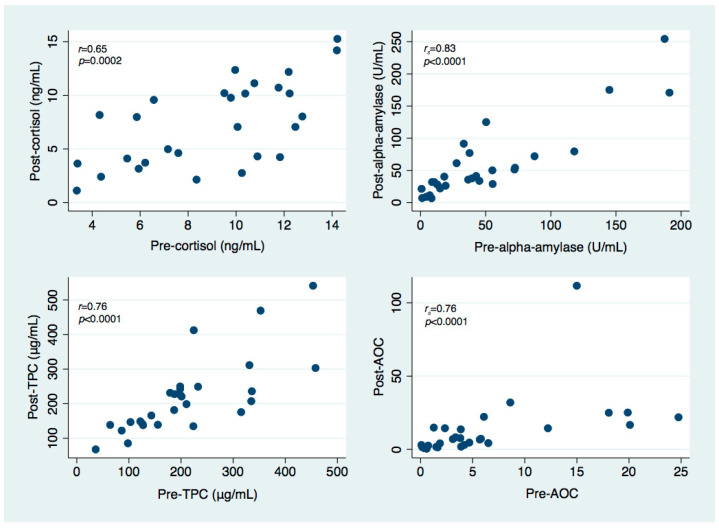
Scatter plots illustrating the relationship between pre-treatment and post-treatment levels of salivary cortisol, salivary alpha-amylase, total protein content (TPC) and salivary alpha-amylase over cortisol ratio (AOC).

For each of the investigated salivary parameters, strong positive correlations were observed between pre-treatment and post-treatment levels, as presented in Figure 1. No relevant correlations were detected between any other salivary parameters.

No statistically significant correlations were observed regarding the relationship between salivary cortisol levels and carious experience indices, number or percentage of untreated dental caries. On the other hand, pre- and post-treatment sAA levels showed statistically significant negative correlations with scores of carious experience indices for primary teeth (dmft and dmfs), while only pre-treatment sAA levels showed a marginally positive correlation with carious experience indices for permanent teeth (DMFT), as depicted in Table 2.

Significant negative correlations were observed between pre-treatment AOC values and dmft (*r_s_* = −0.407, *p* = 0.044, Spearman’s rank correlation coefficient) and dmfs, respectively (*r_s_* = −0.450, *p* = 0.024, Spearman’s rank correlation coefficient). Post-treatment AOC values showed marginal negative correlations with dmft (*r_s_* = −0.384, *p* = 0.058, Spearman’s rank correlation coefficient) and dmfs (*r_s_* = −0.362, *p* = 0.075, Spearman’s rank correlation coefficient). Pre-treatment AOC values showed a significant positive correlation with DMFS (*r_s_* = 0.405, *p* = 0.040, Spearman’s rank correlation coefficient) and a marginal positive correlation with DMFT (*r_s_* = 0.362, *p* < 0.069, Spearman’s rank correlation coefficient), while post-treatment AOC values showed no significant correlations with DMFT and DMFS, respectively (*r_s_* = 0.296, *p* = 0.142 and *r_s_* = 0.317, *p* = 0.115, Spearman’s rank correlation coefficient). Regarding the correlations between AOC ratio and MCL and MCL%, no statistically significant associations were detected. Similarly, pre- and post-treatment values of TPC failed to show any statistically significant correlation with any of the carious experience indices.

All children were assessed as well-behaved during the dental treatments, exhibiting a positive behaviour, corresponding to an FBRS score of 3 (*n* = 8, 28.57%), or a definitely positive behaviour, corresponding to an FBRS score of 4 (*n* = 20, 71.43%). None of the children were uncooperative during the treatment session or unaccepted the treatment. Regarding psychometric anxiety evaluations, mean (SD) normalised T-scores were 49.33 (8.29) for the STAIC S-Anxiety scale and 49.04 (9.70) for the STAIC T-Anxiety scale. The stratification of the children according to their level of anxiety, as delivered electronically by the platform, is presented in Table 3.

Mean (SD) scores for STAIC S-Anxiety assessments registered higher values for children with an FBRS score of 3 (*n* = 8, 57.61 ± 7.02) compared with children with an FBRS score of 4 (*n* = 20, 46.02 ± 6.24), the difference being statistically significant (*p* < 0.001, *t*-test). STAIC T-Anxiety scores did not differ significantly between children with an FBRS score of 3 (*n* = 8, 51.29 ± 10.15) and an FBRS score of 4 (*n* = 20, 48.14 ± 9.63) (*p* = 0.448, *t*-test). No significant correlation was observed between state and trait anxiety assessments (*r* = 0.161, *p* = 0.411, Pearson’s correlation coefficient). Furthermore, no significant correlations were detected between state or trait anxiety scores with neither of pre- or post-treatment levels for salivary cortisol, normalised cortisol, sAA, normalised sAA, TPC or AOC. Age or carious experience did not show any significant correlations to FBRS, STAIC S-Anxiety or STAIC T-Anxiety scores.

The most important findings for the two study groups are presented in Table 4. Children who were subjected to prophylaxis showed a higher decrease in cortisol levels (−2.96 ± 3.43) compared with children subjected to cavity preparation (−0.36 ± 2.13), the difference being statistically significant (*p* = 0.024, *t*-test). Although sAA levels showed a lower increase of post-treatment values for the prophylaxis group (6.80 ± 26.51) compared with the cavity preparation group (12.62 ± 29.25), the difference did not reach statistical significance (*p* = 0.586, *t*-test). Psychometric state and trait anxiety evaluations showed no statistically significant differences between the two intervention groups (*p* > 0.05, *t*-test).

## 4. Discussion

The experience of visiting a dental environment and interactions with the dental team can be stressful for children, especially if their first visit in the dental office takes place in relation to a dental emergency [41]. In order to help a young paediatric patient to better cope during the following treatment sessions, an introductory prophylaxis session is a suitable option to accommodate the child with the dental office and the dental team. Therefore, in our study, in the first treatment session, younger children received prophylaxis, while older children received cavity preparation and a restoration.

Every child is different and will have specific coping reactions. Dentists may incorrectly interpret a cooperative behaviour of children with the lack of stress or anxiety, when in reality patients who seemingly accept the dental procedures might in fact experience psychological stress [42,43]. This suggestion is supported by our results, showing that even though children’s behaviour was evaluated as positive or definitely positive on FBRS assessments, psychometric evaluations revealed that almost two thirds of the subjects scored moderate or high on state (64.28%) and trait (71.43%) anxiety assessments. Children’s behaviour may be unpredictable, especially if they experience dental anxiety. Therefore, it is of utmost importance for the paediatric dentist to be able to quickly and reliably evaluate the child’s possibilities of cooperation during dental treatments in order to adopt the most suitable behavioural approach and conduct the dental treatments accordingly. By doing so, the child might be able to overcome their dental anxiety and have lower stress levels. This might lead to a better cooperation during treatment sessions and might persuade the child to continue to visit the dental office in the future [44].

The levels of salivary cortisol and sAA, registered before and after the dental treatment, had a great variability among our study population. Therefore, we processed statistically both the raw values and the normalised to total protein content levels. The salivary cortisol levels in our study are comparable to reference values of paediatric subjects reported in the literature [45]. The cortisol levels were significantly higher before versus after treatment, suggesting the impact of anticipatory stress. This observation might reside in the fact that subjects were at their first experience in the dental office where the study was conducted, and they were not aware of what could happen. The decreased cortisol levels after completion of the treatment might suggest that the actual experience proved to be less stress-inducing for the subjects than the anticipation.

Furthermore, the decrease in salivary cortisol levels was significantly higher for the prophylaxis group compared with the cavity preparation group, suggesting that non-invasive interventions, such as prophylactic therapy, are less stressful than more complex interventions, which require removal of carious tissue and placement of a restoration. This observation is also supported by the fact that pre-treatment cortisol levels did not show any differences between the two intervention groups, while post-treatment cortisol values recorded differences. Similar results had been observed in other studies, which also reported significantly higher salivary cortisol levels before intervention [46,47]. Furthermore, in older children, salivary cortisol levels did not differ significantly before or after clinical examination [48], confirming that the subject’s age and the complexity of the intervention are important factors associated with dental anxiety in children [41]. This observation might also be related to the fact that older children are more likely to have had previous dental experience, and therefore they might be able to assess what will happen in the dental office. On the other hand, there are studies that report increased salivary cortisol levels as a consequence of dental treatments, with higher salivary levels after complex or pain-inducing interventions, such as cavity restorations and extractions, as opposed to non-invasive procedures, such as routine examinations and prophylaxis [49,50,51,52,53]. In fact, painful treatments are perceived as traumatic dental experiences and are associated with an increased risk of dental anxiety [54].

Our results showed that sAA levels increased post-treatment, but the differences were not statistically significant. Although sAA levels showed a lower increase in post-treatment values for the prophylaxis group compared with the cavity preparation group, the difference did not reach statistical significance. Comparison between groups of both pre- and post-treatment values of sAA did not show significant differences. In addition, before and after treatment variation of sAA observed in our study corresponds to diurnal profile of this biomarker [36], supporting furthermore that non-invasive dental treatments, especially dental prophylaxis, induce minimum stress responses and, therefore, do not influence its variation. Data reported in the literature are still inconclusive regarding the type of stressors related to ANS activation and variations of sAA levels [21,24,55]. Similar to our findings, other studies conducted in children showed increases in sAA levels after various dental treatments, including dental prophylaxis and more invasive procedures, such as extractions [47,56,57]. Conversely, significant anticipatory increases of sAA levels before dental prophylaxis were reported by Furlan et al. (2012) [46]. In addition, Dos Santos et al. (2012) reported that average sAA levels decreased by 41.1% after dental treatment in healthy children [58].

Interestingly, sAA and cortisol levels showed different variations in our study, an observation also reported in the study of Dos Santos et al. (2012) [58]. When exposed to psychological stressors, the ANS system reacted more quickly and sAA displayed a considerable increase in activity, compared with the HPA axis and subsequent salivary cortisol levels [59]. This comes in the context of evidence showing that different stressors activate the two response systems differently, the HPA axis being particularly stimulated by psychological stimuli, while the ANS system is activated by physiological stressors [60]. Therefore, we aimed to minimise bias of physiological stressors by asking the subjects to refrain from physical exercise before saliva collection. In addition, these biomarkers’ levels are differently influenced by changes in salivary flow rate [24]. In order to reduce this potential bias, we collected saliva through a passive drooling method so that the collection method had the least impact on the accuracy of results [61].

Currently, there is a lack of evidence regarding the relationship between dental caries and stress-related modifications of salivary physiology [62], not only that stress leads to modifications of life-style and undesirable behaviours, such as indifference to performing oral hygiene or consuming higher amounts of sugary foods—that have cariogenic effects [63]—but also stress-related alterations of salivary physiology might as well be incriminated [64]. However, as shown by our results, only sAA and AOC levels correlated with carious experience indices, while salivary cortisol showed no influence whatsoever. The differences between groups regarding carious indices for permanent teeth (DMFT and DMFS) are due to age-related differences in dentition development; older children having more time for the permanent teeth to become decayed. Nonetheless, the number or percentage of active carious lesions (MCL and MCL%) did not differ between groups.

Our findings showed no differences between the two intervention groups regarding FBRS or STAIC scores. This suggests that children react differently to various stressors and even non-invasive interventions such as professional brushing with rotary instruments followed by topical fluoridation might be perceived as a stressful event for an anxious patient. For this reason, it is important to correctly evaluate a patient’s anxiety level prior to any dental intervention in order to be able to apply appropriate behavioural management.

We did not find any correlation between state and trait anxiety levels or between any psychometric evaluations and salivary stress marker levels, as opposed to the study of Yfanti et al. (2014), who reported positive correlations between the scores of the two forms of STAIC [50]. In addition, Aoyagi-Naka et al. (2013) found that trait anxiety in children was an important risk factor that caused post-dental treatment increases in sAA levels as a response of ANS [43].

We found that behavioural evaluation scores correlated to results of psychometric evaluations of state anxiety levels, but not with trait anxiety levels. Paradoxically, a study evaluating the perception of paediatric dentists, working in a specialised hospital, regarding the use of dental anxiety scales in their clinical practice revealed increased reluctance in including these evaluations in their day-to-day work, as they failed to see any practical usefulness of this time-consuming step [65]. However, dental anxiety is a complex condition associated with behavioural, cognitive and psychological factors that may not manifest synchronously or evidently [66]. Therefore, for a better comprehension of children’s behaviour during dental treatments, the use of anxiety scales in addition to the dentist’s clinical experience and behavioural managing skills might offer better results.

There are a number of limitations to our study. To further explore the relationship between multiple means of evaluations of dental anxiety and stress induced by dental treatments in children, studies performed on larger populations are required in order to be able to differentiate between different anxiety levels of subjects. The fact that the number of included participants is low is a consequence of the restrictions imposed by the COVID-19 pandemic, which prevented us from recruiting more subjects. We are considering including more subjects in the future. Furthermore, multiple time-point sampling is recommended, since this might offer the possibility to explore the development of patients over time, as well as baseline sampling to set individual reference values. Dissimilarities between our results and the data reported by various other studies might be related to different stressors applied, especially with regard to the complexity of the dental intervention and the degree of discomfort or pain induced [53], as well as to different saliva collection methods and laboratory analysis protocols used in various studies.

Our findings contribute to the existing knowledge of how dental treatments influence the patient’s level of stress. A valuable insight is the demonstration of the interactions of the two stress systems, HPA and ANS, objectively evaluated in this study by measuring salivary levels of cortisol and alpha-amylase. To these physiological assessments, psychological assessments were associated, such as subjective behavioural and objective psychometric evaluations. Furthermore, we aimed to evaluate the influence of stress over carious experience in children. Taken together, our results underline the lack of understanding of the mechanisms through which stress might influence carious experience and how various dental treatments affect children’s behaviour and stress levels.

## 5. Conclusions

Within the limitations of the study, our results showed that the HPA and ANS stress response systems reacted differently to dental treatments. Salivary cortisol decreased, while salivary alpha-amylase increased as a consequence of exposure to a dental treatment. Dental prophylaxis was perceived as less stressful in comparison with cavity preparation (considered a more complex and invasive intervention). Furthermore, including children in a dental education and prevention programme, and starting their dental visits at a younger age, when no dental diseases have already occurred, might be an important strategy to prevent dental anxiety. Age and carious experience did not influence the degree of stress or anxiety levels in children. Children that exhibit less cooperative behaviour during dental treatments had higher levels of state anxiety. Integrating objective and subjective evaluations of children’s behaviour and reactions during dental treatment sessions might enable paediatric dentists to better approach and manage patients’ stress in the dental office.

## Figures and Tables

**Table 1:** Values of salivary parameters evaluated before and after dental treatment.

	Pre-Treatment	Post-Treatment	*p*-Value
	Mean ± SD	Mean ± SD	
Cortisol (ng/mL)	8.99 ± 3.26	7.33 ± 3.94	0.008 ^a^ *
Cortisol normalised (ng/µg protein)	0.06 ± 0.06	0.04 ± 0.03	0.043 ^b^ *
Salivary Alpha-Amylase (U/mL)	50.12 ± 52.50	59.82 ± 57.86	0.572 ^b^
Salivary Alpha-Amylase normalised (U/µg protein)	0.29 ± 0.31	0.28 ± 0.21	0.442 ^b^
AOC	6.43 ± 6.92	13.54 ± 21.16	0.036 ^b^ *
Total Protein Content (μg/mL)	210.12 ± 109.16	218.35 ± 109.13	0.185 ^b^

Cortisol normalised, cortisol to total protein concentration ratio; salivary alpha-amylase normalised, salivary alpha-amylase to total protein concentration ratio; AOC, salivary alpha-amylase over cortisol ratio; *p*, level of significance. ^a^ Paired *t*-test. ^b^ Sign test. * Statistical significance.

**Table 2:** Correlations between pre- and post-treatment levels of salivary alpha-amylase and carious experience.

	Pre-sAA	Post-sAA	Pre-sAA Normalised	Post-sAA Normalised
DMFT	*r_s_* = 0.349	*r_s_* = 0.325	*r_s_* = 0.199	*r_s_* = 0.223
	*p* = 0.080	*p* = 0.105	*p* = 0.329	*p* = 0.273
DMFS	*r_s_* = 0.379	*r_s_* = 0.344	*r_s_* = 0.240	*r_s_* = 0.254
	*p* = 0.056	*p* = 0.086	*p* = 0.237	*p* = 0.210
dmft	*r_s_* = −0.461	*r_s_* = −0.390	*r_s_* = −0.414	*r_s_* = −0.373
	*p* = 0.020 *	*p* = 0.054 *	*p* < 0.040 *	*p* < 0.066
dmfs	*r_s_* = −0.487	*r_s_* = −0.371	*r_s_* = −0.570	*r_s_* = −0.463
	*p* = 0.014 *	*p* = 0.068	*p* < 0.003 *	*p* < 0.020 *
MCL	*r_s_* = −0.236	*r_s_* = −0.169	*r_s_* = −0.280	*r* = −0.211
	*p* = 0.226	*p* = 0.390	*p* = 0.150	*p* = 0.281
MCL%	*r_s_* = −0.299	*r_s_* = −0.191	*r_s_* = −0.286	*r* = −0.186
	*p* = 0.122	*p* = 0.329	*p* = 0.140	*p* = 0.344

sAA—salivary alpha-amylase; DMFT/dmft—decayed-missing-filled per tooth; DMFS/dmfs—decayed-missing-filled per tooth’s surfaces; MCL—manifest carious lesions; MCL%—percentage of manifest carious lesions out of total number of teeth per subject; sAA normalised—sAA to total protein concentration ratio; *r_s_*—Spearman’s rank correlation coefficient value; *p* Level of significance. * Statistical significance.

**Table 3:** Distribution of STAIC S-Anxiety and STAIC T-Anxiety levels.

	STAIC S-Anxiety		STAIC T-Anxiety	
	Frequency	Percentage	Frequency	Percentage
	*n* = 28		*n* = 28	
Low	10	35.71%	8	28.57%
Moderate	8	28.57%	14	50.00%
High	10	35.71%	6	21.43%

**Table 4:** Comparative evaluations for prophylaxis and cavity preparation groups.

	Prophylaxis Group (*n* = 14)	Cavity Preparation Group (*n* = 14)	*p*-Value
	Mean ± SD	Mean ± SD	
Age (years)	6.64 ± 1.20	9.43 ± 1.92	<0.001 ^a^ *
DMFT	1.08 ± 1.51	4.21 ± 3.38	0.009 ^b^ *
DMFS	1.67 ± 2.53	5.93 ± 4.62	0.013 ^b^ *
dmft	7.50 ± 5.53	6.09 ± 2.51	0.442 ^a^
dmfs	20.43 ± 18.80	14.27 ± 6.87	0.968 ^b^
MCL	8.14 ± 5.75	7.57 ± 3.41	0.751 ^a^
MCL%	36.98 ± 25.25	30.76 ± 14.17	0.429 ^a^
Pre-Cortisol (ng/mL)	8.88 ± 2.32	9.11 ± 4.09	0.857 ^a^
Post-Cortisol (ng/mL)	5.92 ± 2.91	8.74 ± 4.41	0.056 ^a^
Pre-sAA (U/mL)	59.52 ± 56.36	40.71 ± 48.55	0.401 ^b^
Post-sAA (U/mL)	66.32 ± 67.64	53.33 ± 47.84	0.571 ^b^
Pre-TPC (μg/mL)	181.04 ± 94.82	239.19 ± 118.05	0.163 ^a^
Post-TPC (μg/mL)	185.70 ± 90.75	251 ± 119.18	0.062 ^b^
Pre-AOC	7.54 ± 7.96	5.32 ± 5.79	0.635 ^b^
Post-AOC	11.72 ± 8.11	15.35 ± 29.28	0.223 ^b^
FBRS	3.71 ± 0.47	3.71 ± 0.47	1 ^c^
STAIC S-Anxiety	48.46 ± 8.86	50.20 ± 7.90	0.589 ^a^
STAIC T-Anxiety	47.80 ± 9.60	50.29 ± 10.00	0.507 ^a^

DMFT/dmft—decayed-missing-filled per tooth; DMFS/dmfs—decayed-missing-filled per tooth’s surfaces; MCL—manifest carious lesions; MCL%—percentage of manifest carious lesions out of total number of teeth per subject; sAA—salivary alpha-amylase; TPC—total protein content; AOC—sAA over cortisol ratio; FBRS—Frankl Behaviour Rating Scale; STAIC S-Anxiety—State–Trait Anxiety Inventory for Children, state questionnaire; STAIC T-Anxiety—State–Trait Anxiety Inventory for Children, trait questionnaire. * Statistical significance. ^a^ Student t-test. ^b^ Mann–Whitney U-test. ^c^ Fisher’s exact test.

## Data Availability

The data presented in this study are available from the corresponding authors upon reasonable request.
